# A Warming World, a Growing Threat: The Spread of Ticks and Emerging Tick-Borne Diseases

**DOI:** 10.3390/pathogens14030213

**Published:** 2025-02-21

**Authors:** Miray Tonk-Rügen, Myriam Kratou, Alejandro Cabezas-Cruz

**Affiliations:** 1 Institute for Insect Biotechnology, Justus Liebig University of Giessen, Heinrich-Buff-Ring 26-32, 35392 Giessen, Germany; 2 Laboratory of Microbiology, National School of Veterinary Medicine of Sidi Thabet, University of Manouba, Manouba 2010, Tunisia; mariem.kratou@hotmail.com; 3 ANSES, INRAE, Ecole Nationale Vétérinaire d’Alfort, UMR BIPAR, Laboratoire de Santé Animale, F-94700 Maisons-Alfort, France

Ticks are obligate hematophagous arachnids that play a critical role in transmitting a wide array of pathogens to humans, domestic animals, and wildlife [1]. As vectors of *Borrelia burgdorferi* sensu lato (Lyme borreliosis), *Anaplasma phagocytophilum* (human granulocytic anaplasmosis), tick-borne encephalitis virus (TBEV), which can cause severe neurological conditions, and Crimean-Congo hemorrhagic fever virus (CCHFV), responsible for a fatal viral disease CCHF, ticks pose significant public health risks worldwide [2,3,4].

*Ixodes ricinus* is the most common tick species in Europe [5] and a vector of *B. burgdorferi*, *A. phagocytophilum*, and TBEV [6,7]. Their survival, reproduction, and seasonal activity are influenced by climatic factors such as temperature and precipitation, making them highly sensitive to environmental changes [8,9]. This sensitivity, combined with their ability to thrive in new environments, raises concerns over the spread of these tick-borne diseases, particularly as global temperatures increase.

In their recent paper, Tsioka et al. [10] provided valuable insights into the diversity of pathogens carried by the *I. ricinus* ticks in a mountainous region of Greece [10]. Their findings highlight the presence of various pathogens such as *B. burgdorferi* and *A. phagocytophilum*, pointing to a significant public health concern for the region [10]. Importantly, in the study, Tsioka et al. [10] identify *I. ricinus* ticks collected from vegetation as the dominant species in a mountainous deciduous forest above 600 m, contrasting with previous studies in Greece [11,12], which focused primarily on ticks from humans or livestock. The studied ecosystem, resembling temperate European habitats where *I. ricinus* is widespread, provides valuable insights into the tick’s ecology and pathogen transmission in this underexplored environment. Notably, the study highlights the impact of geographical and environmental factors, such as high-altitude forest ecosystems, on the prevalence of tick-borne diseases [10]. These ecosystems, with their humid microclimates and diverse wildlife, create optimal conditions for ticks and their pathogens, leading to distinct prevalence patterns shaped by local ecological dynamics.

Climate change significantly impacts the distribution and behavior of vector species, including ticks [13,14,15]. Rising temperatures and altered precipitation patterns are expanding tick ranges into regions previously unsuitable for their survival [16,17,18], while also reducing suitable habitats in areas that become too dry or otherwise unfavorable [10]. For instance, the distribution of *I. ricinus*, typically found in temperate zones, is being significantly altered by these climatic shifts. Warmer temperatures are allowing this species to extend its range into higher-altitude regions and cooler northern latitudes [19,20,21]. The study in Greece, documented the expansion of *I. ricinus* into mountainous regions above 600 m, where deciduous forests provide favorable conditions for the tick [10]. Over the years, the geographical distribution of *I. ricinus* has expanded significantly, with populations now documented in regions such as Scandinavia, as a result of climate change and habitat modifications. Additionally, *I. ricinus* has been found in parts of North Africa [22,23,24], expanding its range beyond the regions where it was previously known to occur [25]. This redistribution of tick populations, rather than a simple increase, may render current epidemiological maps outdated, emphasizing the need for ongoing monitoring and updates to tick-borne disease risk assessments [15]. More importantly, these changes not only alter tick population dynamics but also influence the geographical spread of tick-borne diseases [26,27,28].

However, *I. ricinus* is not the only tick species undergoing distributional changes due to climate change. *Hyalomma marginatum*, increasingly reported in Greece [29,30], is emerging as a significant public health concern across Europe [31,32,33,34]. Recent studies have documented the spread of *H. marginatum* into Europe, facilitated by climate change, increased global trade, and migratory bird pathways. This expansion poses potential risks for the introduction of vector-borne pathogens, highlighting the need for enhanced surveillance in newly colonized areas. Originally native to warmer climates such as the Mediterranean [35], *H. marginatum* is now being reported in previously unrecorded regions, including parts of Germany [36,37,38,39]. This shift is primarily driven by climate change, which has enabled *Hyalomma* ticks to establish northern areas that are typically too cold for their survival. Furthermore, the expansion of *H. marginatum* into northern Europe poses significant public health risks by introducing diseases typically restricted to tropical and subtropical regions, including the CCHFV, a pathogen not commonly found in the area [40,41,42,43].

Additionally, the growing geographic range of *H. marginatum* highlights the urgent need to monitor tick-borne diseases in areas where they were previously rare or absent, as the introduction of these ticks not only increases direct health risks but also complicates disease prevention and control efforts. To address these growing public health challenges, it is crucial for European authorities to adopt proactive strategies, including monitoring tick populations, tracking the spread of diseases like CCHF, and implementing preventive measures such as tick control programs and public awareness campaigns.

In conclusion, the findings of this study emphasize the importance of understanding the role of *I. ricinus* ticks in the transmission of pathogens in Greece, particularly in mountainous areas where these ticks are increasingly prevalent. The effects of climate change on the distribution of ticks, such as *I. ricinus* and *H. marginatum*, highlights the growing threat of tick-borne diseases in new regions. As these ticks expand into previously unaffected areas, they introduce new risks for both humans and animals. Furthermore, the potential for increased transmission of diseases such as Lyme disease, TBE, and CCHF requires urgent attention from public health specialists and the scientific community. Nonetheless, to be prepared and reduce these risks, it is critical for countries to invest in advancing research, enhancing surveillance systems, and implementing comprehensive prevention strategies targeting tick-borne diseases. In addition, public health infrastructures must be strengthened to effectively address emerging vector-borne threats, while educational initiatives are necessary to inform individuals about protective measures against tick exposure. Lastly, climate change is reshaping the ecological landscape, and with it, the dynamics of disease transmission. Proactive strategies, public awareness, and preparedness will be essential to mitigate the impacts of emerging threats and to safeguard public health.
